# Iron overload exacerbates age-associated cardiac hypertrophy in a mouse model of hemochromatosis

**DOI:** 10.1038/s41598-017-05810-2

**Published:** 2017-07-18

**Authors:** Abitha Sukumaran, JuOae Chang, Murui Han, Shrutika Mintri, Ban-An Khaw, Jonghan Kim

**Affiliations:** Department of Pharmaceutical Sciences, Northeastern University, Boston, MA USA

## Abstract

Cardiac damage associated with iron overload is the most common cause of morbidity and mortality in patients with hereditary hemochromatosis, but the precise mechanisms leading to disease progression are largely unexplored. Here we investigated the effects of iron overload and age on cardiac hypertrophy using 1-, 5- and 12-month old Hfe-deficient mice, an animal model of hemochromatosis in humans. Cardiac iron levels increased progressively with age, which was exacerbated in Hfe-deficient mice. The heart/body weight ratios were greater in Hfe-deficient mice at 5- and 12-month old, compared with their age-matched wild-type controls. Cardiac hypertrophy in 12-month old Hfe-deficient mice was consistent with decreased alpha myosin and increased beta myosin heavy chains, suggesting an alpha-to-beta conversion with age. This was accompanied by cardiac fibrosis and up-regulation of NFAT-c2, reflecting increased calcineurin/NFAT signaling in myocyte hypertrophy. Moreover, there was an age-dependent increase in the cardiac isoprostane levels in Hfe-deficient mice, indicating elevated oxidative stress. Also, rats fed high-iron diet demonstrated increased heart-to-body weight ratios, alpha myosin heavy chain and cardiac isoprostane levels, suggesting that iron overload promotes oxidative stress and cardiac hypertrophy. Our findings provide a molecular basis for the progression of age-dependent cardiac stress exacerbated by iron overload hemochromatosis.

## Introduction

Iron is an essential micronutrient in almost all living organisms for various metabolic processes, including oxygen transport, oxidative phosphorylation and neurotransmitter homeostasis^1, 2^. Abnormal iron metabolism due to either iron deficiency or overload results in multiple organ dysfunctions^3^. For example, iron deficiency causes microcytic anemia, cognitive impairment and growth retardation, while excess iron promotes the generation of reactive oxygen species (ROS) and increases oxidative stress that consequently damages parenchymal tissues^3^. Thus, iron levels must be maintained within physiological limits in order to avoid pathological manifestations.

Hereditary hemochromatosis (HH) is an iron overload disorder that occurs primarily due to a deficiency in hepcidin^4, 5^, the principal iron regulatory hormone that maintains systemic iron homeostasis by limiting intestinal iron absorption and iron efflux from the macrophages^6^. While loss of function in several iron modulators (e.g. hepcidin, hemojuvelin, transferrin receptor 2) is associated with decreased hepcidin expression and increased iron absorption^1^, mutations in the HFE (hyperferremia) gene are the most common cause of iron overload hemochromatosis^7^, which affects 0.5% of the North American populations^8^. In addition, iron overload occurs due to repeated blood transfusions in several anemias, including β thalassemia, sickle cell anemia and myelodysplastic syndrome^9^, making iron overload disorders a global health problem^10^.

Since there is no regulated pathway for iron excretion, the absorbed iron progressively accumulates in various organs, including liver, heart and pancreas, leading to liver cirrhosis, cardiomyopathy, diabetes and arthritis^11^. In particular, cardiomyopathy is the most common cause of morbidity and mortality in patients with iron overload disorders^9, 12, 13^. For instance, Engle *et al*. described the prevalence of iron overload associated cardiomyopathy and heart failure in patients with thalassemia^14^. Moreover, cardiovascular diseases contribute to the morbidity and mortality of patients with HH^15, 16^. In contrast, some studies indicate no association between cardiac dysfunction and hemochromatosis^17, 18^. Carpenter *et al*. showed that there is no evidence for hypertrophy in patients with β thalassemia and hereditary hemochromatosis, when compared with healthy subjects^17^. These findings suggest that iron overload alone may not promote cardiac damage and that other factor(s) could increase the susceptibility to the progression of cardiovascular disorders associated with iron loading.

In iron overload disorders, iron uptake by the heart is much slower than that by the liver, and thus cardiac iron accumulation occurs much later than hepatic iron overload^19, 20^. While iron is stored in the heart as ferritin, excessive or labile iron can promote the formation of ROS, resulting in tissue damage and subsequent organ failure^19^. Importantly, iron stores increase with age^21^, and it has been well-documented that aging contributes to increased oxidative stress^22–24^. For example, elevated ROS by the aged mitochondria along with concomitant decreases in the antioxidant defense mechanisms promote the damage of various tissues, including the heart^25^. Several studies have also shown a strong association between oxidative stress and the development of cardiac hypertrophy^26, 27^. These lines of evidence suggest that iron overload could increase age-associated oxidative stress and predispose to cardiac damage.

Despite a large body of evidence that patients with iron overload suffer from heart-related problems, the precise mechanisms of cardiotoxicity in iron overload hemochromatosis is not clearly understood. The present study was aimed at characterizing the underlying mechanisms involved in cardiac hypertrophy in iron overload hemochromatosis using Hfe-deficient mice, a mouse model of HH in humans, and to evaluate the influence of age on the disease progression. The role of iron loading in the development of cardiac hypertrophy was also verified by a rat model of dietary iron overload. Our results provide important evidence that individuals with iron overload HH could be more susceptible to age-associated cardiac stress, likely due to increased ROS mediated by elevated iron stores in the heart.

## Materials and Methods

### Animal care and procedures

Breeders of Hfe-deficient (*Hfe* ^−/−^)^28–30^ and wild-type (*Hfe* ^+/+^) control mice on the 129S6*/*SvEvTac background were kindly provided by Dr. Nancy Andrews (Duke University Medical Center, NC). The *Hfe* ^−/−^ mice display the same iron loading HH phenotype observed in humans^28–35^. Weanling mice were fed facility chow (250 mg iron/kg diet) and water *ad libitum*. To examine the effect of age on iron-related cardiac stress, age-matched male *Hfe* ^−/−^ and *Hfe* ^+/+^ mice (1, 5 and 12 months of age) were used for the study. Mice were euthanized by isoflurane overdose, followed by exsanguination and the removal of heart and liver. To examine the effect of iron overload on cardiac hypertrophy without an influence of Hfe deficiency, an animal model of dietary iron overload was included: weanling Sprague-Dawley rats were fed iron overload diet (10,000 mg carbonyl iron/kg diet; Harlan Teklad) or basal diet (50 mg iron/kg diet) for 5 weeks, followed by euthanasia for tissue collection. All experiments were performed in strict accordance with the recommendations in the Guide for the Care and Use of Laboratory Animals of the National Institutes of Health. The protocol was approved by the Northeastern University Animal Care and Use Committee.

### Non-heme iron analysis

Liver and heart tissues were incubated in a 15-fold volume of acid solution (10% trichloroacetic acid, 3 M HCl) in 65 °C water bath for 20 h. Samples (0.08 mL) were mixed with a reaction buffer (10% thioglycolic acid and 1% bathophenanthroline disulfonic acid in saturated sodium acetate) for colorimetric reaction^36^. Serum iron was determined as previously described^36^. The optical density was measured using UV/Vis spectrophotometer at 535 nm. Non-heme iron concentration was determined based on serially-diluted iron standard solutions. Data were presented in ppm (i.e. µg iron per gram of wet tissue weight).

### Fluorescence microscopy

Heart tissues were cryopreserved using tissue freezing medium (OCT). Tissue sections (10 µm) were fixed with acetone and stained with bispecific anti-myosin antibody^37^, followed by incubation with a polymer which was conjugated to anti-dithiopropionic acid-rhodamine isothiocyanate. The samples were then imaged using fluorescence microscopy (Nikon Eclipse E400). Polymer controls were performed for all of the fluorescence microscopy experiments to confirm the specificity of detection. The relative degree of myosin expression was evaluated by four researchers who were blinded to the experiments, based on the rank order of fluorescence signal intensity of all individual microscope images. The combined scores of the signal intensities were used to quantify and compare the differences among the groups.

### Western blot analysis

Snap-frozen heart tissues were homogenized in RIPA buffer (50 mM Tris, 0.1% SDS, 1% NP-40, 0.5% sodium deoxycholate, pH 7.5) containing protease inhibitors (Complete Mini, Roche) with 0.5 mM phenylmethanesulfonylfluoride. Tissue homogenates were centrifuged at 16,000 *g* for 6 min at 4 °C. Protein concentrations in heart homogenates were determined by the Bradford assay. The tissue extracts (20–80 µg protein) were electrophoresed on 10% gels and transferred to nitrocellulose membranes for 150 mA for 3 h. The membranes were incubated with blocking solution (0.05% Tween 20, 5% non-fat milk in TBS) for 1 h at room temperature, followed by incubation with primary antibodies in 2% non-fat milk at 4 °C for overnight. Antibodies used were mouse anti-alpha myosin heavy chain (AMHC, 1:500, Abcam) and mouse anti-beta myosin heavy chain (BMHC, 1:1,000, Sigma-Aldrich). Blots were probed with mouse anti-actin (MP Biomedicals) as a loading control. Secondary antibodies were sheep anti-mouse antibodies (GE Healthcare). Immunoreactivity was detected using ECL West Dura substrate (Thermo Scientific). Protein bands were visualized by ChemiDoc XRS (Bio-Rad) and intensities of protein bands were quantified using Image Lab (version 4.1, Bio-Rad).

### Histopathological analysis

Heart tissues from 12-month old *Hfe* ^−/−^ mice and their age-matched wild-type mice were fixed in 4% formalin, paraffin-embedded and sectioned into thin slices (5 µm), which were mounted on microscope slides for H&E and Masson’s trichrome staining.

### Real-time qPCR

RNA was isolated from snap-frozen tissues of *Hfe* ^−/−^ and *Hfe* ^+/+^ mice using TRI reagent (Sigma-Aldrich) as per the manufacturer’s instructions. RNA (1 µg) was reversely transcribed into cDNA, which was used for real-time polymerase chain reaction assays. The iScript^TM^ reverse transcription supermix and iTaq^TM^ universal SYBR® green supermix were obtained from Bio-Rad, USA. Primers for the nuclear factor of activated T-cells (NFAT) c1, c2, c3, and c4^38^ were obtained from Eurofins, MWG Operon. The expression levels of the NFAT subtypes were normalized to those of β-actin to compare the relative expression of NFAT subtype mRNA levels between *Hfe* ^−/−^ and *Hfe* ^+/+^ mice.

### Isoprostane analysis

Free isoprostane levels in the heart tissues were measured using 8-isoprostane EIA assay kit (Cayman Chemical). Briefly, tissues (20–30 mg) were homogenized in 0.1 M Tris buffer (pH 7.4) with 1 mM EDTA and 0.005% butylated hydroxytoluene. The homogenates were centrifuged at 8,000 *g* for 10 min at 4 °C. The supernatant was further diluted with EIA buffer and loaded into the strips pre-coated with mouse anti-rabbit IgG. The tracer (acetylcholinesterase linked to 8-isoprostane) and antiserum to 8-isoprostane were added to the samples in the wells and incubated for 18 h at 4 °C. After wash, the color was developed by incubating the plate in Ellman’s reagent for 2 h and the absorbance was measured with a spectrophotometer at 410 nm. Concentrations of isoprostane were calculated based on the standard curve.

### Statistical analysis

Values reported were expressed as means ± SEM. Two-way ANOVA was employed to assess the effects of age and the *Hfe* gene, as well as interaction effects (age x *Hfe* gene), followed by the Tukey’s post-hoc analysis for pairwise comparisons (Systat; version 13). Differences were considered significant at p < 0.05.

## Results

### *Hfe*^−/−^ mice display cardiac hypertrophy with age

The body weight progressively increased in an age-dependent manner in both *Hfe* ^+/+^ and *Hfe* ^−/−^ mice (Table 1). However, there were no significant differences in the body weight between *Hfe* ^−/−^ mice and their age-matched wild-type controls. Liver and heart weights also significantly increased as the age increased. Notably, *Hfe* ^−/−^ mice displayed significantly greater heart-to-body weight ratios at 5-month old (12% increase, p = 0.022) and 12-month-old (15% increase, p = 0.003) when compared with their age-matched wild-type counterparts (Table 1). These results indicate a significant association between Hfe deficiency and age in cardiac hypertrophy. The liver-to-body weight ratios were greater in 12-month-old *Hfe* ^−/−^ mice compared with age-matched *Hfe* ^+/+^ controls. Combined, these results demonstrate that loss of Hfe function results in enlarged heart and liver with age.

**Table 1:** Physiological characteristics of Hfe-deficient mice at 1, 5 and 12 months of age.

	N	1-month		5-month		12-month	
		*Hfe* ^+/+^	*Hfe* ^−/−^	*Hfe* ^+/+^	*Hfe* ^−/−^	*Hfe* ^+/+^	*Hfe* ^−/−^
Body weight^†^ (g)	8–12	14.5 (0.4)	15.6 (0.3)	27.4 (0.5)	26.6 (0.5)	34.4 (1.3)	33.4 (0.4)
Heart weight^†,#,^^ (g)	8–12	0.081 (0.002)	0.079 (0.001)	0.124 (0.003)	0.135 (0.003)	0.162 (0.009)	0.187* (0.005)
Liver weight^†,#^ (g)	8–12	0.62 (0.04)	0.73 (0.02)	1.03 (0.05)	0.98 (0.03)	1.10 (0.06)	1.25* (0.02)
Heart/Body weight^†,^^ (%)	8–12	0.56 (0.02)	0.51* (0.01)	0.45 (0.01)	0.51* (0.01)	0.49 (0.02)	0.56* (0.01)
Liver/Body weight^†,#^ (%)	8–12	4.26 (0.19)	4.68* (0.10)	3.77 (0.15)	3.67 (0.10)	3.20 (0.15)	3.76* (0.08)

Data are presented as the means (SEM). Two-way ANOVA was employed to assess the main effects (age and *Hfe* gene) as well as interaction effects (age x *Hfe* gene), followed by the Tukey’s post-hoc analysis for pairwise comparisons (Systat; version 13).

^†^p < 0.05, age effect.

^#^p < 0.05, *Hfe* effect.

^^^p < 0.05, age x *Hfe* effect.

*p < 0.05, *Hfe* ^−/−^ vs. age-matched *Hfe* ^+/+^ mice.

### Hfe deficiency elevates iron in the heart with age

**Figure 1:**
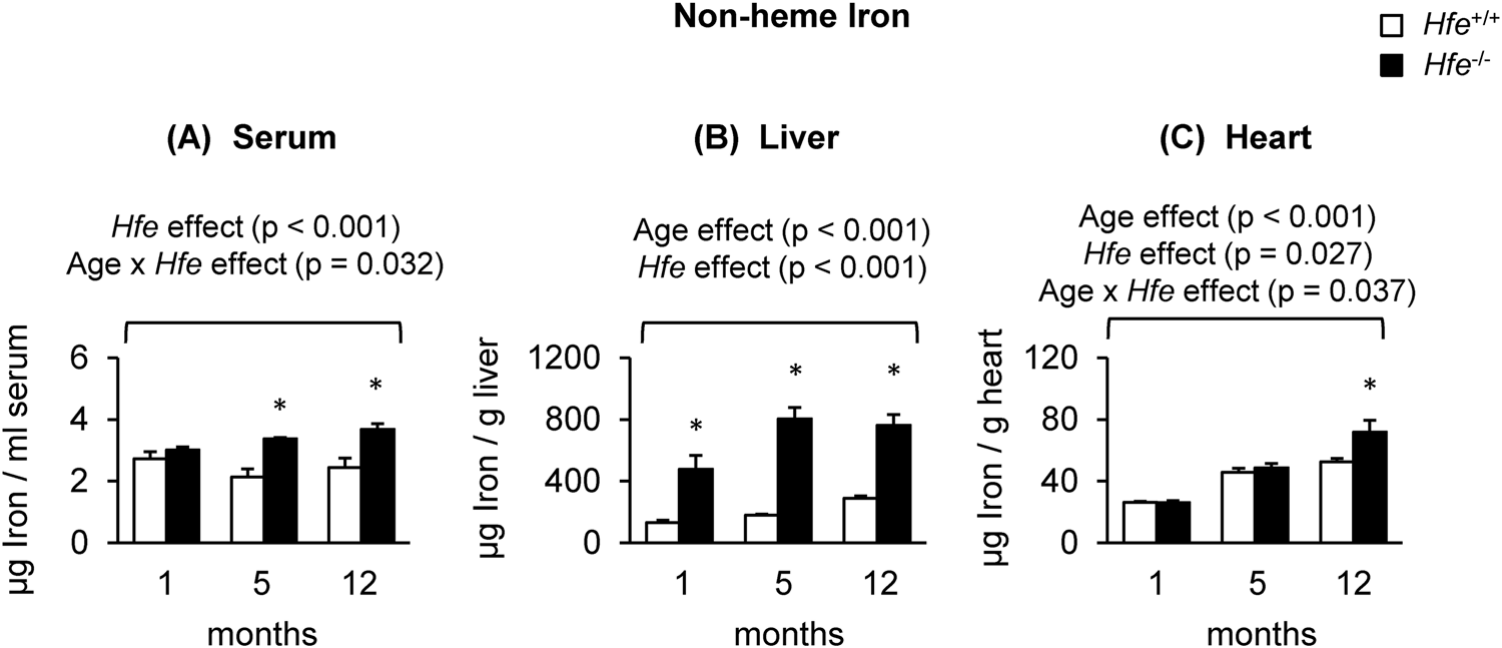
Non-heme iron levels in serum, liver and heart of *Hfe* ^+/+^ and *Hfe* ^−/−^ mice. Non-heme iron content in serum (**A**), liver (**B**) and heart (**C**) was measured colorimetrically using bathophenanthroline (n = 5–9 per group). Data are presented as the means ± SEM. *p < 0.05 between *Hfe* ^−/−^ and *Hfe* ^+/+^ mice assessed by the two-way ANOVA, followed by the Tukey’s post-hoc analysis.

Levels of serum iron were significantly higher in *Hfe* ^−/−^ mice than in their age-matched wild-type controls at 5-months (p < 0.001) and 12-months old (p < 0.001), but not at 1-month old (Fig. 1A), suggesting a gradual iron loading in the circulation over time in the absence of Hfe. The liver non-heme iron content gradually increased with age in both *Hfe* ^+/+^ and *Hfe* ^−/−^ mice, but was significantly increased in *Hfe* ^−/−^ mice at all ages studied compared with their age-matched wild-type controls (Fig. 1B). There was an age-dependent, progressive increase in the steady-state levels of iron in the heart, regardless of Hfe expression, suggesting that cardiac iron content increases with age (Fig. 1C). Moreover, cardiac non-heme iron levels were significantly higher in *Hfe* ^−/−^ mice at the age of 12 months (36% increase; p < 0.001), but not of 1 month or 5 months, when compared with age-matched wild-type mice (Fig. 1C). These results demonstrate that the heart becomes loaded with iron with age, which is exacerbated in Hfe deficiency.

### Myosin levels are increased in the heart of *Hfe*^−/−^ mice

**Figure 2:**
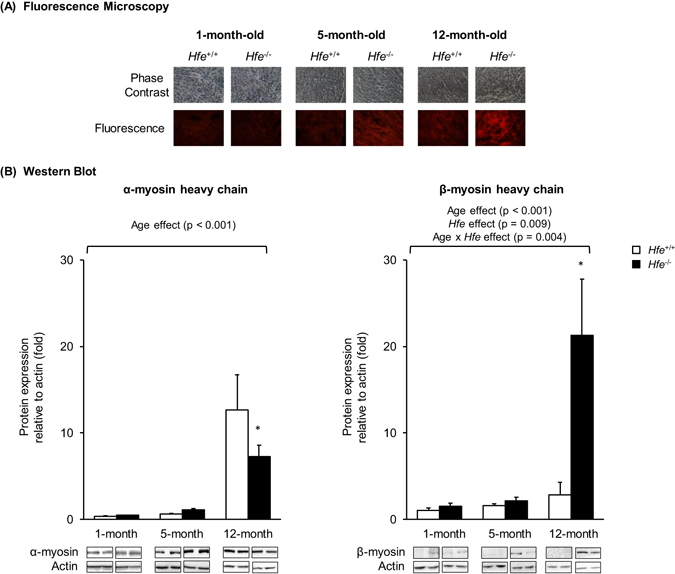
Effect of age on the expression of cardiac myosin heavy chains in *Hfe* ^+/+^ and *Hfe* ^−/−^ mice. (**A**) Cardiac myosin expression in 1-month, 5-month and 12-month old *Hfe* ^+/+^ and *Hfe* ^−/−^ mice (n = 3–5 per group) was evaluated by fluorescence microscopy. Frozen heart tissues were sectioned using cryostat (10 µm thickness) and stained with bispecific myosin antibody polymer, which was conjugated to anti-dithiopropionic acid-rhodamine isothiocyanate. Sections stained without bispecific polymer were used as background control. (**B**) Representative immunoblots are shown for α- and β-myosin heavy chains in heart tissues of 1-month, 5-month and 12-month old *Hfe* ^+/+^ and *Hfe* ^−/−^ mice (n = 4–6 per group). The bar graph represents the relative expression in the protein level of α- and β-myosin heavy chains, normalized to that of actin. Data are presented as the means ± SEM. *p < 0.05 between *Hfe* ^−/−^ and *Hfe* ^+/+^ mice assessed by the two-way ANOVA, followed by the Tukey’s post-hoc analysis.

Our observation of cardiac hypertrophy upon Hfe deficiency in older ages prompted us to determine the levels of α- and β-myosin heavy chains, indicative of cardiac hypertrophy, in the heart of these mice. We first characterized the expression levels of cardiac myosins by fluorescence microscopy using heart cryosections; myosin levels appeared to be increased in 5-month and 12-month old mice, but not in 1-month old mice, upon Hfe deficiency (Fig. 2A). This reflects an increased expression of myosins by loss of Hfe function with age. To obtain a more quantitative interpretation, we employed western blot analysis and found significantly lower levels of α-myosin heavy chains (42% decrease) and increased levels of β-myosin heavy chain expression in 12-month old *Hfe* ^−/−^ mice (653% increase) (Fig. 2B). Taken together, our results show that increased cardiac hypertrophy in Hfe deficiency is associated with elevated expression of myosin heavy chains in the heart and that there is a shift in cardiac myosin expression from α- to β-myosin heavy chains as the age increases.

### Cardiac fibrosis is increased in *Hfe*^−/−^ mice

**Figure 3:**
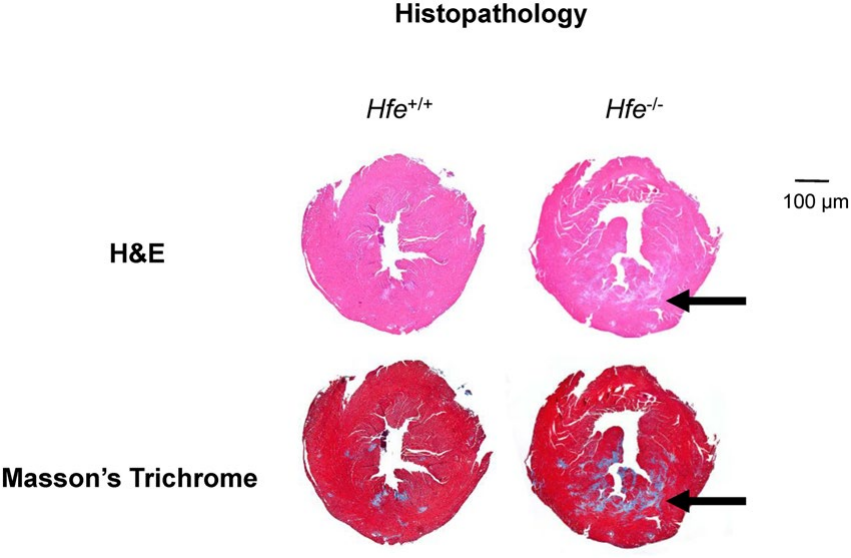
Cardiac fibrosis in *Hfe* ^+/+^ and *Hfe* ^−/−^ mice. Heart tissues from 12-month old *Hfe* ^−/−^ mice and their age-matched wild-type mice were fixed in 4% formalin, embedded in paraffin and sectioned into 5 µm thin slices, which were mounted on microscope slides for H&E and Masson’s trichrome staining. Arrows indicate the areas of fibrosis.

Since 12-month old *Hfe* ^−/−^ mice showed signs of cardiac hypertrophy along with increased iron content, heart tissues from 12-month old animals were further examined for histopathological characteristics. H&E and Masson’s trichrome staining demonstrated increased cardiac fibrosis in *Hfe* ^−/−^ mice compared with age-matched *Hfe* ^+/+^ mice (Fig. 3).

### NFAT-c2 expression is increased in *Hfe*^−/−^ mice

**Figure 4:**
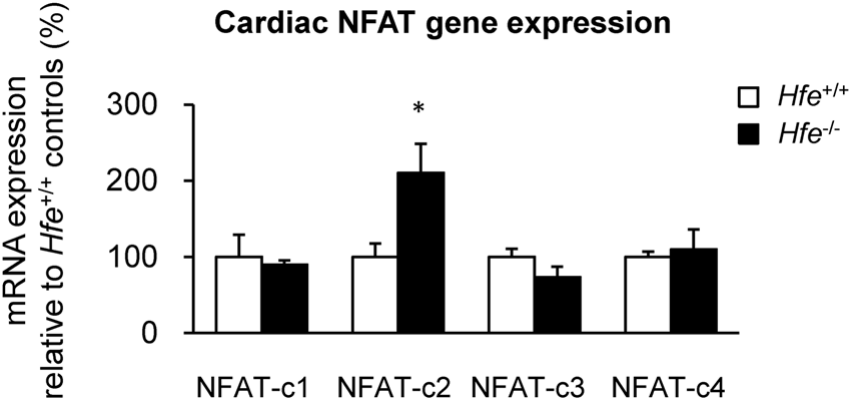
NFAT expression in *Hfe* ^+/+^ and *Hfe* ^−/−^ mice. The expression levels of the NFAT genes in the heart from *Hfe* ^+/+^ and *Hfe* ^−/−^ mice (n = 5 per group) at 12-months of age were quantified by real-time qPCR. Data are presented as the means ± SEM. *p < 0.05 between *Hfe* ^−/−^ and *Hfe* ^+/+^ mice assessed by the two-sample *t*-test.

To explore the role of cardiac iron and age in the progression of cardiac hypertrophy and up-regulation of cardiac myosin heavy chains, we compared mRNA levels of NFAT, a marker of cardiac hypertrophy, in the heart from *Hfe* ^−/−^ and *Hfe* ^+/+^ mice at the age of 12-month old. While there were no significant changes in the expression of NFAT-c1, NFAT-c3 or NFAT-c4 between *Hfe* ^−/−^ and *Hfe* ^+/+^ mice, the levels of NFAT-c2 were significantly up-regulated in *Hfe* ^−/−^ mice (Fig. 4).

### Cardiac isoprostane levels are increased in *Hfe*^−/−^ mice

**Figure 5:**
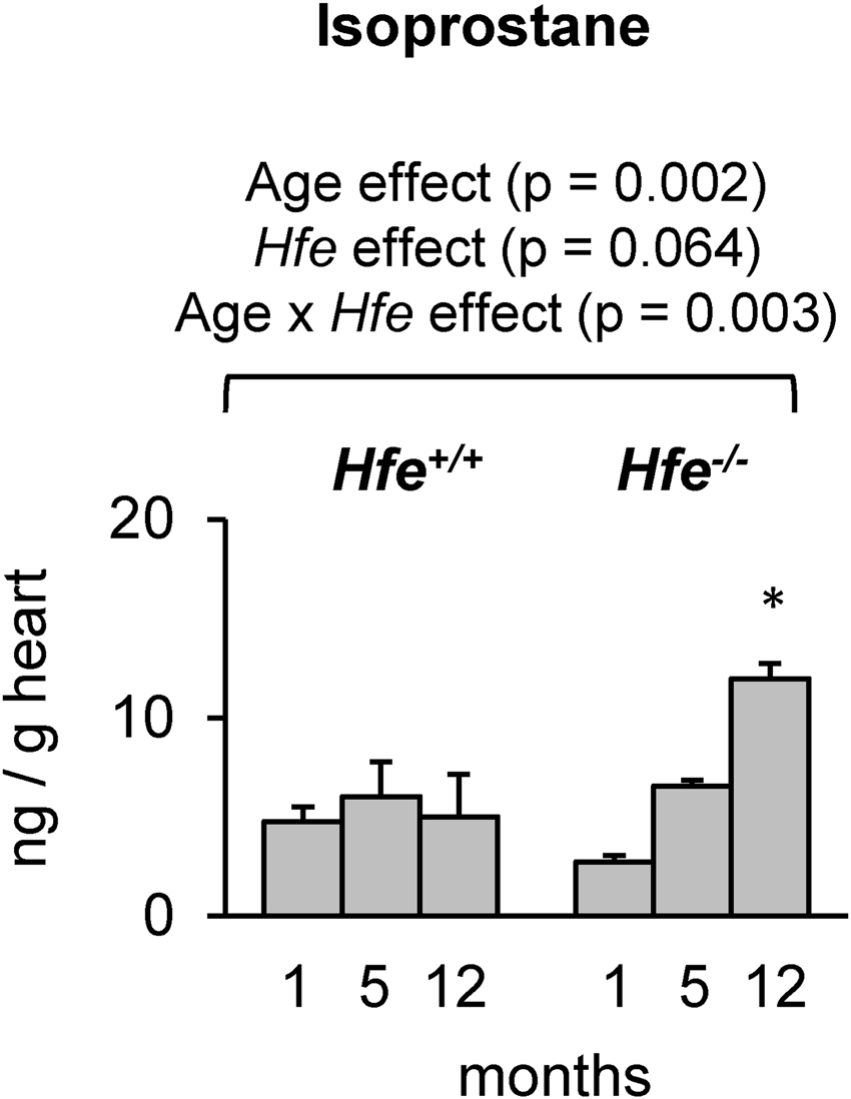
Levels of isoprostane in *Hfe* ^+/+^ and *Hfe* ^−/−^ mice. Levels of isoprostane in the heart of *Hfe* ^+/+^ and *Hfe* ^−/−^ mice (n = 5 per group) at 1, 5, and 12 months of age were determined by 8-isoprostane kit. Data are presented as the means ± SEM. *p < 0.05 between *Hfe* ^−/−^ and *Hfe* ^+/+^ mice assessed by the two-way ANOVA, followed by the Tukey’s post-hoc analysis.

Levels of isoprostane, a marker of oxidative stress, were not different among all age groups in *Hfe* ^+/+^ mice. However, *Hfe* ^−/−^ mice showed a progressive increase in cardiac isoprostane levels with age (Fig. 5). In addition, isoprostane levels were significantly greater in *Hfe* ^−/−^ mice at 12 months of age compared with age-matched wild-type mice, indicating elevated oxidative stress in the heart with age in iron overload hemochromatosis.

### Dietary iron overload promotes cardiac hypertrophy

**Figure 6:**
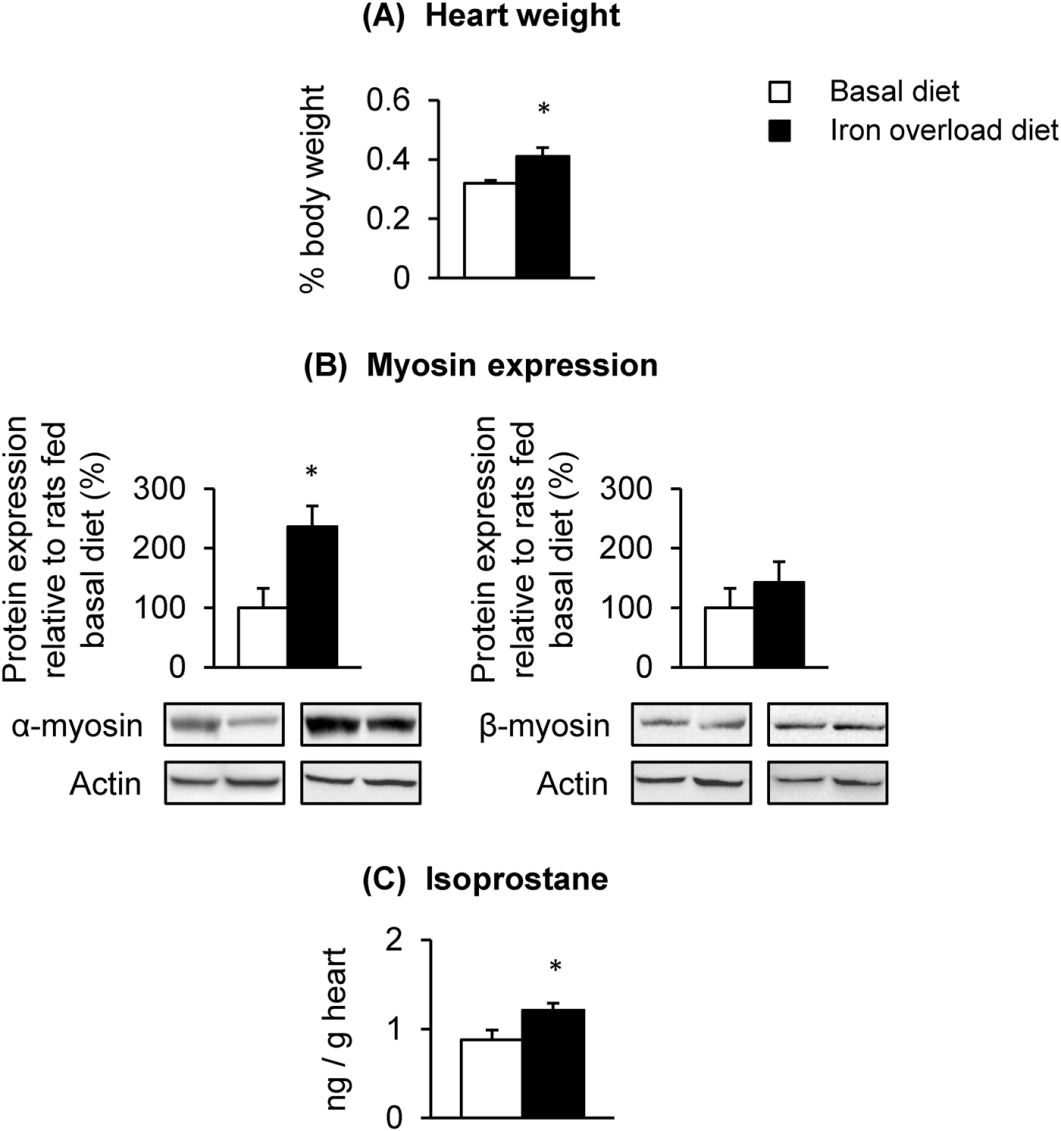
Effect of dietary iron overload on cardiac hypertrophy and oxidative stress in rats. Sprague-Dawley rats were fed basal diet (50 mg iron/kg diet) or iron overload diet (10,000 mg carbonyl iron/kg diet) for 5 weeks (n = 4 per group) to determine the heart-to-body weight ratios (**A**), levels of α- and β-myosin heavy chains (**B**) and isoprostane levels (**C**) in the heart. Data are presented as the means ± SEM. *p < 0.05 between rats fed basal diet and those fed iron overload diet assessed by the two-sample *t*-test.

To examine if cardiac hypertrophy in *Hfe* ^−/−^ mice resulted from iron loading in the heart or from a potential direct effect of Hfe deficiency, we used rats fed iron overload diet. Consistent with the results from genetic iron overload animals (i.e. *Hfe* ^−/−^ mice), rats fed iron overload diet displayed increased heart/body weight ratios (Fig. 6A; p = 0.003), which was associated with an up-regulation of α-myosin heavy chains (Fig. 6B, p = 0.031). Again, isoprostane levels were increased in rats fed with iron overload diet (Fig. 6C, p = 0.032). Combined, these data support an idea that iron loading promotes cardiac hypertrophy and potentially increases vulnerability to heart injury in iron overload hemochromatosis.

## Discussion

In the present study, we explored the effects of iron loading and age on cardiac hypertrophy. Since HFE-related hemochromatosis represents the most prevalent iron overload disorder^7^, we used *Hfe* ^−/−^ mice with three different age groups: 1, 5 and 12 months of age. We observed an age-dependent increase in the heart-to-body weight ratio in both *Hfe* ^+/+^ and *Hfe* ^−/−^ mice. Moreover, the increase was greater in Hfe deficiency, which was associated with increased cardiac iron content and oxidative stress, as well as elevated levels of cardiac β-myosin heavy chains and fibrosis. To our knowledge, this is the first study to define the role of cardiac iron in age-related cardiac hypertrophy in a mouse model of hemochromatosis. Our findings suggest that elevated cardiac iron due to hemochromatosis could increase the age-associated risks of cardiovascular diseases in mice.

Consistent with previous studies^29, 33, 39^, our *Hfe* ^−/−^ mice showed increased levels of serum and liver iron, when compared with their age-matched wild-type mice. Notably, cardiac iron content was significantly elevated in *Hfe* ^−/−^ mice only at 12 months of age, but not at earlier time points (1 and 5 months old), suggesting that iron accumulation in the heart occurs more slowly than that in the liver^19, 20^. In contrast, Miranda *et al*.^40^ have shown elevated cardiac iron content in *Hfe* ^−/−^ mice at 8 weeks of age. This difference could result from several factors that influence iron transport and metabolism, including differences in sex and dietary iron content, as well as different methods of measurement of cardiac iron. For example, we used male mice fed standard facility chow containing 250 mg iron/kg diet, whereas Miranda *et al*. studied female mice with no information available on dietary iron content. In addition, we employed bathophenanthroline assay to measure “non-heme” iron content^36^, while Miranda *et al*. used atomic absorption spectrophotometry, which determines both heme and non-heme iron. It remains to be elucidated the effects of Hfe on heme iron metabolism in relation to cardiac function. While the mechanism by which cardiac iron stores are increased in hemochromatosis is not completely understood, several iron transporters, including the transferrin receptor, divalent metal transporter 1, ZRT/IRT-like protein 14 (Zip14), have been implicated in cardiac iron homeostasis^41, 42^. Also, a recent report demonstrated that an iron exporter ferroportin plays an important role in cardiac iron homeostasis^43^. In addition, studies have shown a potential role of L-type Ca^2+^ channels in iron transport in the heart^44, 45^. Further studies are warranted to investigate the molecular mechanism of cardiac iron transport in iron overload disorders and the effect of age on the expression of these transporters. Combined, our study suggests that Hfe deficiency increases iron levels in the circulation and hepatic iron stores, and later promotes iron accumulation into the heart.

The heart requires a large supply of energy, and thus, cardiomyocytes are rich in mitochondria and consume large amounts of oxygen^46^. However, cardiomyocytes have decreased levels of antioxidant enzymes compared with other organs^47^. Therefore, increased demands for oxygen along with inappropriately low levels of antioxidant enzymes make the heart highly susceptible to oxidative injury^46^. Importantly, iron in excess acts as a catalyst in the formation of toxic hydroxyl radicals from superoxide anion and hydrogen peroxide, resulting in the damage of macromolecules, such as DNA and proteins^48^, and organ dysfunction^49, 50^. Consistent with this idea, we found elevated levels of isoprostane, which are prostaglandin-like substances produced as a result of peroxidation of arachidonic acid by free radicals^51^, in 12-month old *Hfe* ^−/−^ mice. Notably, *Hfe* ^+/+^ mice displayed increased cardiac iron with no change in isoprostane levels as the age progresses, suggesting the existence of protective mechanism(s) against age-associated oxidative stress in the presence of Hfe. Together, our study affirms that there is an age-dependent increase in cardiac oxidative stress in iron overload, which could contribute to the development of cardiac hypertrophy and thereby increase the risk of cardiovascular diseases in the elderly.

Since myocardial remodeling occurs during stress conditions^52^, we explored if loss of Hfe function could alter the expression of α and β myosin heavy chains, which are abundantly expressed in the heart and serve as the molecular motors. The α myosin heavy chain (AMHC) has a higher ATPase activity when compared with the β myosin heavy chain (BMHC) and thus the contractile velocity of the heart is proportional to the relative levels of AMHC and BMHC^53^. The AMHC accounts for >90% of total myosin heavy chains in the rodent heart^54–56^. In the present study, we found that *Hfe* ^−/−^ mice have decreased cardiac AMHC and increased BMHC at 12-months of age, suggesting an AMHC-to-BMHC transition in the production of myosin heavy chains. Studies have shown changes in the expression levels of AMHC and BMHC under stress^53, 57, 58^. Increased BMHC is the hallmark of cardiac hypertrophy^59^. Moderate induction of mean aortic pressure significantly increases the expression of BMHC in rats^54^. Previous reports suggest that an increase in BMHC could be an adaptive response for the optimum function of contraction/relaxation cycle^60^, but the underlying molecular mechanisms are poorly understood. Importantly, studies in humans and rats have also shown that the levels of BMHC increase as the age progresses^53, 61^. Therefore, age-associated up-regulation of BMHC, which was even greater in Hfe deficiency and correlated with cardiac iron, could predispose to cardiac hypertrophy and other types of cardiovascular diseases. Combined, our study provides important evidence that cardiac iron loading can accelerate the natural aging process of the heart, especially cardiac hypertrophy and fibrosis, and potentially heart failure, which occurs in several iron overload disorders (e.g. hemochromatosis, thalassemia and sickle cell anemia)^9–11^. Further, our study suggests an importance of therapeutic intervention to reduce cardiac iron in a timely manner.

To further understand the molecular mechanism of cardiac hypertrophy in *Hfe* ^−/−^ mice, we measured mRNA expression of cytoplasmic subunits of the NFAT transcription complex. Calcineurin/NFAT signaling has been shown to have a variety of functions in different tissues. A recent study showed that NFAT-c2 is implicated in calcineurin-mediated myocyte hypertrophy^62^. Our finding of elevated NFAT-c2 expression upon Hfe deficiency suggests that increased cardiac iron could activate the calcineurin-NFAT signaling pathway, which promotes cardiac hypertrophy^63, 64^. Further studies are necessary to better understand the mechanism by which iron regulates the calcineurin/NFAT signaling pathway and initiates hypertrophic responses. While NFAT-c1 is involved in regulating the expression levels of genes for heart valve development^65^, we did not observe any significant changes in the mRNA levels of the NFAT-c1 gene in *Hfe* ^−/−^ mice. Unlike NFAT-c1 and c2, c3 and c4 are involved in the transcriptional activation of many genes in lymphocytes and in the brain, respectively^65^. Together, our study suggests an important role of the NFAT-c2 gene in the development of cardiac hypertrophy, likely via the calcium-dependent signaling pathway. Alternatively, iron overload could enhance the expression of atrial natriuretic peptide/brain natriuretic peptide and/or influence hypertrophic events mediated by proinflammatory cytokines (e.g. TNFα and IL-6)^66^. Future studies are warranted to investigate the role of Hfe in the regulation of these events.

Since loss of Hfe function results in iron overload, we attempted to separate iron loading effect from a genetic influence of Hfe deficiency on cardiac hypertrophy by treating rats with high iron diet for 5 weeks. We used rats rather than mice since several reports have demonstrated that mice fed iron overload diet fail to show a significant increase in heart iron^67^ or cardiac hypertrophy^68^, whereas rats with dietary iron overload display increased cardiac iron and heart damage^41, 69^. These studies indicate that rats are a relevant rodent model to evaluate the role of acquired iron loading in the progression of cardiac hypertrophy. Our results demonstrated that dietary iron overload in rats increases heart-to-body weight ratios along with elevated isoprostane levels, supporting the idea that cardiac hypertrophy and oxidative stress in *Hfe* ^−/−^ mice are likely induced by iron loading, and not by a direct/intrinsic effect of Hfe deficiency. Moreover, iron overload induced by high iron diet for 5 weeks was correlated with increased AMHC content, but not BMHC, suggesting that prolonged exposure to high iron (e.g. nutrition, age, Hfe mutation, transfusion) could promote an AMHC-to-BMHC transition and heart damage. It remains to be explored how genetic alternations (Hfe deficiency) and external stimulus (iron loading) trigger the conversion of AMHC to BHMC.

The present study defines a significant role of age-associated cardiac iron loading in HFE-related hemochromatosis. Our results provide an improved understanding of the pathogenesis of cardiac hypertrophy, which will contribute to the development of novel therapeutic strategies for the treatment of iron overload-associated cardiomyopathy.
